# Supplementary material for: Hot spot prediction in protein-protein interactions by an ensemble system
Source: BMC Syst Biol. 2018 Dec 31;12(Suppl 9):132. doi: 10.1186/s12918-018-0665-8 (PMC6311905; doi:10.1186/s12918-018-0665-8)
Supplement: Supplementary file 2 — All relevant MATLAB code. A simple Matlab implement of our predictor. (ZIP 19,742 kb) [file 12918_2018_665_MOESM2_ESM.zip › Additional file 2/Read ASA/ASA Property/SKEMPI/1TM1I.txt]

 NetSurfP - Protein Surface Accessibility and Secondary Structure Predictions

  

|  |  |  |
| --- | --- | --- |
|  |  | NetSurfP - Protein Surface Accessibility and Secondary Structure Predictions  Technical University of Denmark |

  


---

```
# For publication of results, please cite:
# A generic method for assignment of reliability scores applied to solvent accessibility predictions.
# Bent Petersen, Thomas Nordahl Petersen, Pernille Andersen, Morten Nielsen and Claus Lundegaard
# BMC Structural Biology 2009, 9:51 doi:10.1186/1472-6807-9-51
#
# Column 1: Class assignment - B for buried or E for Exposed - Threshold: 25% exposure, but not based on RSA
# Column 2: Amino acid
# Column 3: Sequence name
# Column 4: Amino acid number
# Column 5: Relative Surface Accessibility - RSA
# Column 6: Absolute Surface Accessibility
# Column 7: Z-fit score for RSA prediction
# Column 8: Probability for Alpha-Helix
# Column 9: Probability for Beta-strand
# Column 10: Probability for Coil
E M  1TM1I                  1    0.755 151.055  -0.135   0.016   0.005   0.979
E K  1TM1I                  2    0.501 102.953   0.011   0.053   0.043   0.903
E T  1TM1I                  3    0.530  73.539  -0.265   0.113   0.087   0.800
E E  1TM1I                  4    0.455  79.471   0.832   0.184   0.043   0.773
B W  1TM1I                  5    0.189  45.527   0.465   0.257   0.016   0.727
B P  1TM1I                  6    0.309  43.833  -0.071   0.430   0.016   0.555
E E  1TM1I                  7    0.539  94.216  -0.573   0.502   0.102   0.396
B L  1TM1I                  8    0.156  28.527   0.047   0.538   0.173   0.289
B V  1TM1I                  9    0.191  29.403   0.982   0.428   0.171   0.402
E G  1TM1I                 10    0.356  27.986  -0.830   0.125   0.227   0.648
E K  1TM1I                 11    0.320  65.865   1.130   0.056   0.142   0.802
E S  1TM1I                 12    0.422  49.470   1.140   0.052   0.084   0.864
B V  1TM1I                 13    0.162  24.823   0.283   0.938   0.007   0.055
E E  1TM1I                 14    0.635 110.987   0.694   0.970   0.001   0.030
E E  1TM1I                 15    0.391  68.395   1.585   0.970   0.001   0.030
B A  1TM1I                 16    0.020   2.226   0.686   0.970   0.001   0.030
E K  1TM1I                 17    0.269  55.313   1.735   0.970   0.001   0.030
E K  1TM1I                 18    0.496 102.048   1.732   0.975   0.003   0.022
B V  1TM1I                 19    0.216  33.276   2.271   0.938   0.007   0.055
B I  1TM1I                 20    0.036   6.697   0.656   0.938   0.007   0.055
E L  1TM1I                 21    0.440  80.527   1.437   0.802   0.014   0.185
E Q  1TM1I                 22    0.631 112.679   0.982   0.522   0.016   0.462
E D  1TM1I                 23    0.447  64.442   0.614   0.257   0.016   0.727
E K  1TM1I                 24    0.348  71.584  -0.079   0.058   0.017   0.925
E P  1TM1I                 25    0.307  43.577  -0.709   0.018   0.019   0.964
E A  1TM1I                 26    0.512  56.389   0.642   0.004   0.085   0.910
B A  1TM1I                 27    0.031   3.471   0.342   0.004   0.514   0.481
E Q  1TM1I                 28    0.320  57.134   1.663   0.001   0.900   0.099
B I  1TM1I                 29    0.025   4.699   0.907   0.001   0.959   0.040
B I  1TM1I                 30    0.165  30.543   1.133   0.001   0.959   0.040
B V  1TM1I                 31    0.057   8.715   0.596   0.001   0.959   0.040
B L  1TM1I                 32    0.114  20.910   1.347   0.001   0.900   0.099
B P  1TM1I                 33    0.205  29.061   0.079   0.004   0.514   0.481
E V  1TM1I                 34    0.523  80.447  -0.166   0.005   0.015   0.979
E G  1TM1I                 35    0.699  55.035  -2.404   0.018   0.019   0.964
B T  1TM1I                 36    0.229  31.707  -0.861   0.019   0.141   0.840
E I  1TM1I                 37    0.380  70.300  -1.086   0.022   0.359   0.619
B V  1TM1I                 38    0.235  36.104   0.813   0.021   0.451   0.528
B T  1TM1I                 39    0.213  29.585   0.212   0.022   0.359   0.619
E M  1TM1I                 40    0.463  92.726   0.112   0.020   0.205   0.775
E E  1TM1I                 41    0.451  78.825  -1.460   0.020   0.205   0.775
B Y  1TM1I                 42    0.118  25.174   0.858   0.021   0.279   0.699
E R  1TM1I                 43    0.309  70.807   0.398   0.005   0.262   0.733
B I  1TM1I                 44    0.104  19.277  -0.008   0.004   0.085   0.910
E D  1TM1I                 45    0.272  39.238  -0.025   0.004   0.085   0.910
B R  1TM1I                 46    0.094  21.412   0.887   0.003   0.718   0.279
B V  1TM1I                 47    0.017   2.613   1.203   0.001   0.959   0.040
B R  1TM1I                 48    0.052  11.885   1.698   0.001   0.959   0.040
B L  1TM1I                 49    0.014   2.600   1.587   0.001   0.959   0.040
B F  1TM1I                 50    0.038   7.687   1.000   0.001   0.959   0.040
B V  1TM1I                 51    0.033   5.087   0.370   0.001   0.900   0.099
B D  1TM1I                 52    0.097  13.978   0.520   0.005   0.336   0.660
E R  1TM1I                 53    0.658 150.796  -1.190   0.005   0.015   0.979
E L  1TM1I                 54    0.675 123.666  -0.307   0.003   0.003   0.994
B D  1TM1I                 55    0.208  30.030  -0.575   0.005   0.045   0.951
E N  1TM1I                 56    0.288  42.149   1.197   0.004   0.420   0.576
B I  1TM1I                 57    0.088  16.298  -0.091   0.002   0.816   0.182
B A  1TM1I                 58    0.224  24.630   0.065   0.001   0.900   0.099
E Q  1TM1I                 59    0.410  73.226   0.251   0.001   0.900   0.099
B V  1TM1I                 60    0.197  30.340   0.657   0.004   0.420   0.576
B P  1TM1I                 61    0.278  39.491  -0.275   0.004   0.197   0.799
E R  1TM1I                 62    0.513 117.523  -0.239   0.005   0.262   0.733
E V  1TM1I                 63    0.344  52.934  -0.532   0.004   0.197   0.799
E G  1TM1I                 64    0.864  68.013  -1.991   0.003   0.003   0.994

Explain the output.  Go back.
```

---
